# The Current Safety Regulation for Radiation Emergency Medicine in Korea

**DOI:** 10.3390/ijerph182312434

**Published:** 2021-11-26

**Authors:** You Yeon Choi, Seung Yeol Yoo, Mihyun Yang, Ki Moon Seong

**Affiliations:** National Radiation Emergency Medical Center, Korea Institute of Radiological & Medical Sciences, Seoul 01812, Korea; c9640@kirams.re.kr (Y.Y.C.); ysy@kirams.re.kr (S.Y.Y.); mhy@kirams.re.kr (M.Y.)

**Keywords:** safety regulation, emergency medicine system, radiation, licensees, inspectors

## Abstract

Radiation emergency medicine (REM) systems are operated around the world to provide specialized care for injured individuals who require immediate medical attention in accidents. This manuscript describes the current status of REM safety regulation in Korea and summarizes an assessment of the effects of this regulation. Responding to the requests of people for stronger safety regulations related to radiation exposure, a unique REM safety regulation for nuclear licensees, which is enforceable by laws, has been established and implemented. It is not found in other countries. It can provide a good example in practice for sustainable REM management including document reviews on medical response procedures and inspections of equipment and facilities. REM preparedness of nuclear or radiologic facilities has been improved with systematic implementation of processes contained in the regulation. In particular, the medical care system of licensees has become firmly coordinated in the REM network at the national level, which has enhanced their abilities by providing adequate medical personnel and facilities. This legal regulation service has contributed to preparing the actual medical emergency response for unexpected accidents and should ultimately secure the occupational safety for workers in radiation facilities.

## 1. Introduction

After the Fukushima Daiichi radiation accident, public concerns and anxiety about radiation exposure from the nuclear power plants operating on the Korean peninsula have increased and led to the development of stronger safety regulations for radiation exposure [1,2]. Regulating nuclear and radiation safety is a national responsibility to protect people and the environment from the harmful effects of ionizing radiation. Many governments adopt the International Atomic Energy Agency (IAEA)’s safety standards for use in their national regulations [3]. The Nuclear Safety and Security Commission (NSSC), a governmental authority for nuclear safety and usage regulations in Korea, has also endeavored to strengthen emergency preparedness and the response system for radiation disasters, including emergency medical responses [4,5].

**Figure 1:**
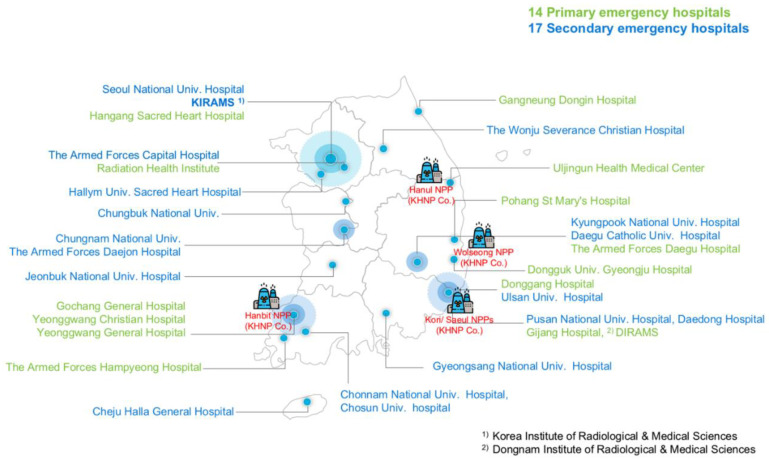
Radiation emergency medicine hospital network and nuclear power plant (NPP) in South Korea (as of June 2021). This network consists of 14 primary emergency hospitals and 17 secondary emergency hospitals, with the National Radiation Emergency Medical Center (NREMC) as the control center. The figure is modified from the available data on the website [9].

Among other strategies, the NSSC established the national radiation emergency medicine network. This network consists of 14 primary emergency hospitals and 17 secondary emergency hospitals, with the National Radiation Emergency Medical Center (NREMC) as the control center (Figure 1). When a radiation accident occurs, primary hospitals should immediately dispatch trained medical staff and provide appropriate emergency medical treatment either at the accident site or at their hospital. Severely injured patients are transferred to the secondary hospitals, which provide advanced medical support, including a large number of beds available for mass-casualty situations [6,7]. Operation and management of the emergency medical network are facilitated by coordinated communication between emergency hospitals and NREMC, together with NSSC. In addition, NSSC safety regulations for radiation emergency medicine (REM) have been expanded to include nuclear or radiologic facilities. First medical response at accident sites is a very important factor for optimizing the efficient treatment of injured individuals [7]. In this instance, injured victims in a radiation accident are planned to be transported from the affected nuclear facilities to designated hospitals in cooperation with an emergency medical transport system, consisting of the National Fire Agency and the other transport agencies registered in the regional health center. Regular exercise on radiation emergency medical response is implemented annually including transportation of the injured in REM system in Korea.

As stated in the legal Article 45 of the Act on Physical Protection and Radiological Emergency, all nuclear licensees, should establish medical preparedness plans for human protection and care of injuries in a radiation emergency, as well as implement drills at their facilities following these plans [8]. This paper describes the current REM safety regulation system for nuclear licensees in Korea and summarizes the current assessment for the system operation.

## 2. Review and Inspection of Radiation Emergency Medicine Plans

As entrusted by NSSC, the REM plans of nuclear or radiologic facilities are legally examined by inspectors who complete an authorized course for inspection in REM. These inspectors are experts with extensive knowledge and experience regarding REM. They have to take the specialized course for education & training of review for four weeks and inspection with a final qualifying examination. These inspectors are officially registered and managed by NSSC. The plans contain practical procedures for rescuing injured individuals, administering first aid, and transferring patients to hospitals, based on the IAEA guidance [10,11]. Inspectors and the licensee develop detailed response procedures in emergency situations, including clearly described methods of patient classification, decontamination, initial treatment, and transportation to the designated hospital, together with the installation of essential equipment and supplies. To strengthen the patient transfer system, the plans contain collaboration and coordination processes with NREMC, together with various agencies such as fire stations, emergency rescue services, emergency hospitals, police, and the Korean armed forces [4,12]. These response plans are created in accordance with international standards (primarily from IAEA) [13,14,15], as well as by considering the individual facility’s characteristics (Table 1). For example, specialized first aid procedures and medical professionals are included in the plans for nuclear facilities in IAEA category I and II, whereas they are not included in the plans for category III facilities.

**Figure 2:**
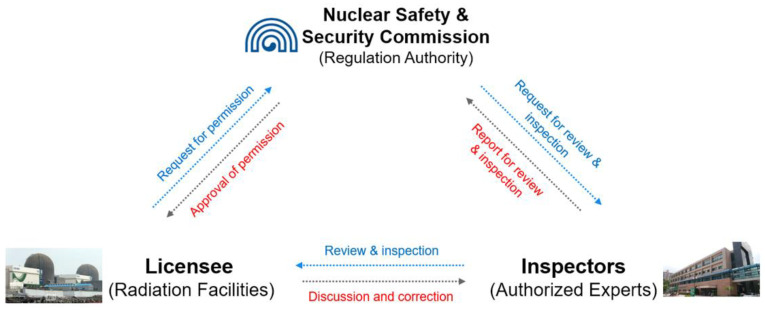
Safety regulation of radiation emergency medicine for nuclear licensees.

NSSC provides two regulatory services related to the REM plan of nuclear licensees (Figure 2). These include review related to approval of the REM plan and inspection of the plan based on the entrustment pursuant to Article 45 of the Act on Physical Protection and Radiological Emergency and Article 40 of Enforcement Degree of the Act [16,17]. Each process is briefly described as follows:

### 2.1. Review Related to Plan Approval

When it is necessary to develop or revise a plan, as may occur when a plan for new facilities has been developed or when correction demands have arisen from an inspection, a nuclear licensee submits a request to NSSC for legal approval of them. NSSC then sends a request for review of the plan to an authorized agency. Based on the legal Article 45 above mentioned [16], the approval for REM plans is determined at 90 days and 60 days for the review on newly developed and revised plans, respectively. For a decision to approve a REM plan, the review results should be turned in to NSSC within at least 1 week prior to the fixed period. When completed, the authorized agency submits a review report to NSSC. NSSC then decides whether to approve the plan and notifies the nuclear licensee of the result.

### 2.2. Inspection

All of the licensee’s facilities are inspected on-site for REM preparedness before the nuclear facilities begin operation, and periodic inspection is conducted once every 1 or 2 years, depending on the type of facility. In addition to regular inspections, some nuclear facilities can be specially inspected when safety issues related to REM emerge, such as when there are changes in the facilities or relevant regulations. The inspection plan should encompass all REM-related facilities, equipment, and staff in the nuclear licensee. Insufficiencies identified during an inspection are shared with the licensee, leading to a discussion between the inspectors and licensees regarding how to fill the gaps. If there are any issues in the inspection report that fail to meet the REM safety regulation criteria, NSSC will request that the licensee complete the appropriate corrective or supplementary measure, based on the inspection report.

## 3. Communications among Stakeholders for Successful Safety Regulation of Radiation Emergency Medicine in Korea

The REM safety regulation has resulted in an annual average of 5.75 reviews and 21 inspections for 27 facilities from 2017 to 2020 in Korea. In accordance with the Act, inspectors examine the REM plan of the licensee. The plan should contain an optimized scale of people and organizations responsible for REM, as well as clearly described job profiles, education, and training [18]. All emergency medical staff should be properly protected from radiation exposure during REM events. Adequate facilities and equipment should be prepared for safe and efficient performance of REM, including personal protective equipment (PPE), radiation decontamination facilities, communication devices, therapeutic agents, and related medical supplies, such as stretchers, splints, defibrillators, ventilators, oxygen, and ambulances. All of these are carefully reviewed in the plan, checking the specific items designated in the Act; the items are inspected on-site at the facilities. Detailed procedures for radiation emergencies are also reviewed [12,19,20].

In the initial period after the law came into effect, licensees had some difficulties with creating an emergency medical plan because they had insufficient experience and reference materials. Their plans often contained limited systems for transferring injured people to the local hospital, as well as insufficient procedures for rescue and first aid. Through communications with stakeholders related to the REM safety regulation, inspectors requested the assignation of a licensee’s emergency response team to these medical preparedness jobs, which would make the REM system of licensees sustainable.

Since the REM safety regulation was implemented, REM preparedness of nuclear or radiologic facilities has improved by the systematic operation in the national network. In the event of a radiological disaster, emergency injuries are most likely to arise at the nuclear facility where the accident occurred. These nuclear facilities are equipped for the REM system with items such as medical personnel, facility, and supplies that enable prompt initial emergency treatment for a certain period of time without external support. In particular, the medical care system for injured people has become firmly established, enabling individuals to be transferred to designated hospitals at the national level beyond the licensee’s own capacity, which can be coordinated by NREMC [4]. The ability to provide medical care for injured patients has also been improved through continuous implementation of the REM safety regulation related to review and inspection. However, some aspects require further improvement on the sustainable management of REM systems, including consideration of long-term and recovery phases, management of thyroid blocking agents, management of disposable medical supplies with expiry dates, and the education and training of assistants to aid with REM activities.

## 4. Evaluation on the Radiation Emergency Medicine Regulatory System

**Figure 3:**
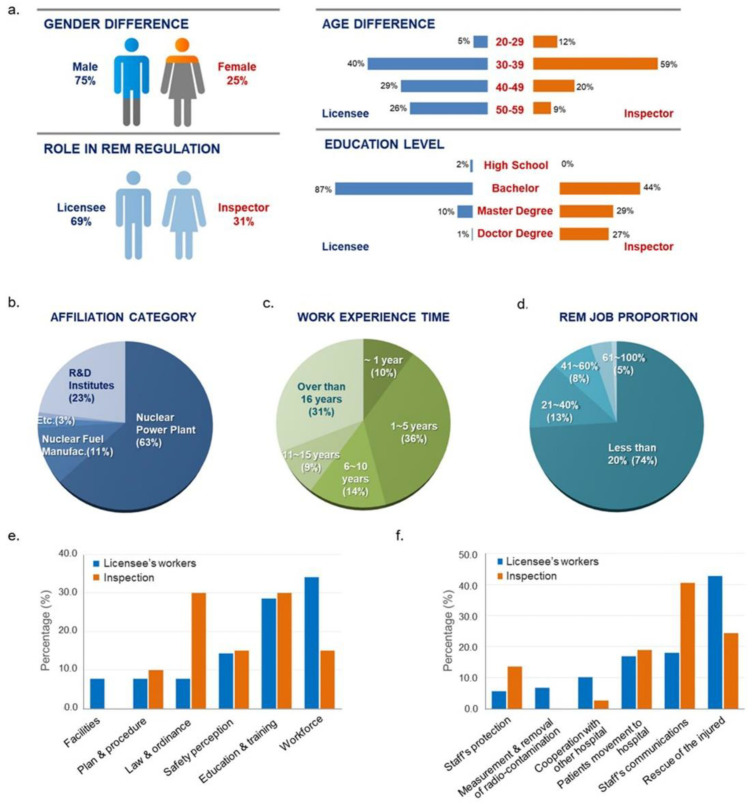
Important factors for safety regulation of radiation emergency medicine. (**a**) General characteristics of respondents, (**b**) Distribution of the affiliation types, (**c**) Distribution of career period (**d**) Job proportion in REM, (**e**) Important factors of REM safety regulation, (**f**) Important factors in the response of REM. REM: regulation of emergency.

The REM regulatory system was recently evaluated using a questionnaire survey of licensee workers and inspectors involved in the creation of REM plans [21]. Among the distributed 150 questionnaires for REM regulatory system, 133 responses were collected (88.7%). Some responses were returned in person and the others were delivered by e-mail as a scanned file to increase the response rate for the survey. All the questionnaires were anonymously answered. General characteristics of respondents were shown in Figure 3a. They are safety officers, administrators, medical doctors, and medical researchers, including health physicists, radiation biologists, and medical specialists. Approximately 70% of the respondents were radiation workers affiliated with companies and institutes undergoing a revision and inspection of the emergency medicine plan, and 63% of them worked for a nuclear power company (Figure 3b). They have degrees in their majored fields, including Bachelor’s (98/133), Master’s (21/133), and Doctoral (12/133) degrees, and over half of them had more than six years of experience with radiation emergency preparedness (Figure 3c). The respondents thought that the national radiation emergency medical system was well prepared, and that the respective emergency medical system of each licensee was sufficiently effective to protect people from a radiation crisis in Korea. They also agreed that the emergency medical system is appropriately managed for radiation safety and enforced by laws (3.53 ± 0.7 on a 5-point Likert scale). These positive perceptions could be considered the consequence of persistent efforts and investments for radiation safety since the Fukushima nuclear power plant accidents [22]. An average of 2.6 million dollars have been invested annually since 2016, and approximately 700 people were involved in building the REM safety regulation system. Furthermore, 41 specially trained inspectors examine the REM plans of all licensees on a regular basis and conduct on-site inspections during radiation accident drills.

In the survey, opinions regarding the important factors of REM safety regulation differed between inspectors and licensee workers. The licensee workers considered the workforce for emergency medicine as being very necessary for radiation safety, whereas legal regulation was considered a very low priority. By contrast, the inspectors considered laws and ordinances to be the most important factors for an efficient, sustainable response of the emergency medical system (Figure 3e). Additionally, licensee workers perceived that the rescue activity was the highest priority for the radiation emergency medical response, unlike inspectors, who prioritized communication with the emergency staff (Figure 3f). These differing views could be explained by their roles in the regulatory system. For most licensee workers, less than 40% of their work was related to radiation emergency preparedness, such that preparedness may have been considered an additional, minor aspect of their work (Figure 3d). Job proportion could be considered a contributing factor influencing implementation of required tasks in emergency situations [23,24,25]. Assigning workers to solely emergency medicine tasks should be considered to secure a robust system of radiation safety regulation.

## 5. Risk Perception as a Critical Factor for Planning and Responses to Radiation Emergencies

Risk perception has been increasingly recognized as a significant factor influencing emergency planning and responses around the world [26,27]. Several studies have shown that risk perception is crucial in emergency responses, as it can directly influence the care that medical personnel provide in nonconventional disasters, such as Ebola virus outbreaks, SARS outbreaks, and the COVID-19 pandemic [28]. Currently, the COVID-19 pandemic offers an opportunity to show that crisis perception and risk communication is critical to support efforts in responding and mitigating a public health crisis. Risk perception based on the reliable information on COVID-19 has given medical staff a strong sense in combatting COVID-19 [29]. Indeed, accurate communication between emergency medical staff has been reported to be necessary for rapid, effective response system to cope with potential disasters [30]. In addition, risk perception of emergency medical staff regarding radiation exposure can strongly influence the creation of safety regulation guidelines, as well as the inspection of the licensees’ emergency plans [31,32]. A survey of radiation risk perception of emergency medical staff in Korea, including occupational and medical care-related radiation exposure, revealed lower risk perception for low-level exposure of radiation [20,30]. Less than 10% of respondents thought that low-level radiation exposure occurred in occupational and medical care and could cause health problems. Considering the occurrence of occasional small accidents involving patients in Korea, even with very low exposure doses (below several mSv) [33], we can anticipate proactive performance of REM staff during emergencies. However, the response of medical staff could be affected by the extent of acceptable radiation use, as well as their perception of radiation exposure risk in daily life [34]. Interestingly, the level of radiation knowledge has been an important factor associated with risk perception of medical staff, which was a finding seen in other groups of individuals as well. Based on the above observations, it is reasonable to assume that continuous education and training for radiation emergencies, including improving radiation knowledge, could lead to a high level of voluntary REM response that is sufficient to meet the requirements of the safety regulation.

## 6. Conclusions

The legal regulation service in Korea has contributed to the preparation of an actual medical emergency response for unexpected accidents which should ultimately secure the occupational safety of workers in radiation facilities. In particular, the regulation provides licensees with the legal basis for obtaining facilities, equipment, and manpower for medical emergency responses, which are necessary to maintain their capabilities for rescuing, applying first aid, and transporting injured individuals. Furthermore, it assists licensees with delivering care from the accident site to the hospital, such as through provision of a medical consultant for thyroid protection, a system to obtain support supplies, and arrangements for transporting patients to the hospital through connections with the national emergency medicine network. This REM safety regulation enforceable by laws is a unique system in Korea, which is not found in other countries. It may be a useful model for radiation safety regulations in other areas of the world.

Similar to the medical preparedness and response system for radiation emergencies in Korea, a nuclear emergency medical network in Japan was re-established in 2018. According to their own legal Article of Nuclear Emergency Act, the Nuclear Regulation Authority (NRA) of Japan developed an Emergency Preparedness and Response (EPR) guide, which proposes how to develop medical systems for a nuclear disaster. Five radiation emergency medical support centers are involved in the medical systems, including the National Institutes for Quantum and Radiological Science and Technology, Hirosaki University, Fukushima Medical University, Hiroshima University, and Nagasaki University, together with many nuclear emergency hospitals and cooperative medical institutes. The NRA supported the emergency facilities and equipment for the medical systems responding to a radiation disaster. Japanese radiation emergency medical systems would be well operated if they are continuously managed well without any interruption. It provides an environment for accepting radiation-exposed patients, as well as related education and training through close coordination between participated organizations. Unfortunately, the REM network in Japan does not include the legal safety regulation system in the same way as the Korean REM safety regulation [35,36]. In view of the efficient and sustainable management of the radiation emergency medical system implemented in Korea, it may be better if preparation and inspection of REM plans are applied to this network and enforced by laws.

Our evaluation of REM staff on the safety regulation and risk perception explained the different opinions depending on their actual properties of roles in REM system and their knowledge level regarding radiation. Understanding their different perceptions could lead to finding an efficient way to improve the regulatory system for radiation emergency medicine. There were some limitations in the evaluation. It was the first time the REM regulation was assessed without a comparison with previous data and external evaluation. To get more valuable estimation of REM regulation, surveys with expanded respondents are strongly recommended to be performed every two or three years, including non-radiation experts and international experts of REM. Many opportunities should be provided to exchange the opinions about safety regulation and the actual management of REM systems, including technical workshops and social meetings. These could contribute to the enhanced performance of REM staff in case of a radiation disaster by increasing their basic knowledge on radiation exposure and reducing the gap in the stakeholders of REM system.

## Figures and Tables

**Table 1:** Radiation facilities in Korea (June 2021).

Licensee	Type	Radiation Facility	Facility Scale ^(1)^	IAEA Category ^(^^2)^
Korea Hydro and Nuclear Power Co. (KHNP)	Power reactor	Nuclear power plants (Kori, Saeul, Wolsong, Hanul, Hanbit)	Large	I
Korea Atomic Energy Research Institute (KAERI)	Research reactor	Hanaro	Large	Ⅱ
	Fuel fabrication facility	Saebit fuel science building	Large	Ⅲ
	Fuel recycling	Fuel testing facilities	Small	
	Waste processing	Inflammable waste processing facility	Small	
KEPCO Nuclear Fuel (KNF)	Nuclear fuel manufacturing		Small	
Korea Radioactive Waste Agency (KORAD)	Radioactive waste storage, processing facilities	Radioisotope waste processing facility	Small	
		Low- and medium-levelradioactive processing facility		
Soyagreentech Co.	Industrial isotope facility	Large-scale irradiating facility	Small	
Greenpia Technology Co.	Industrial isotope facility	Large-scale irradiating facility	Small	

^(1)^ Based on the Act on Physical Protection and Radiological Emergency in Korea; ^(2)^ International Atomic Energy Agency General Safety Requirements No. GSR Part 7.

## Data Availability

Data is contained within the article.
